# Neural-humoral responses during head-up tilt in healthy young white and black women

**DOI:** 10.3389/fphys.2014.00086

**Published:** 2014-03-04

**Authors:** Sara S. Jarvis, Shigeki Shibata, Yoshiyuki Okada, Benjamin D. Levine, Qi Fu

**Affiliations:** ^1^Institute for Exercise and Environmental Medicine, Texas Health Presbyterian Hospital DallasDallas, TX, USA; ^2^Department of Internal Medicine, University of Texas Southwestern Medical CenterDallas, TX, USA; ^3^Department of Biological Sciences, Northern Arizona UniversityFlagstaff, AZ, USA

**Keywords:** muscle sympathetic nerve activity, renin, aldosterone

## Abstract

Young black women have higher prevalence of hypertension during pregnancy compared to white women, which may be attributable to differences in blood pressure (BP) regulation. We hypothesized that young normotensive black women would demonstrate augmented muscle sympathetic nerve activity (MSNA) and renal-adrenal responses to orthostasis. Fifteen white and ten black women (30 ± 4 vs. 32 ± 6 years; means ± SD) had haemodynamics and MSNA measured during baseline (BL), 30 and 60° head-up tilt (HUT), and recovery. Blood was drawn for catecholamines, direct renin, vasopressin, and aldosterone. BL brachial systolic BP (SBP: 107 ± 6 vs. 101 ± 9 mmHg) and diastolic BP (DBP: 62 ± 4 vs. 56 ± 7 mmHg) were higher in white women (both *p* < 0.05). Δ DBP (60° HUT-BL) was greater in black women compared to white (*p* < 0.05). Cardiac output and total peripheral resistance were similar between groups. MSNA burst frequency was higher in whites (BL: 16 ± 10 vs. 14 ± 9 bursts/min, main effect *p* < 0.05) and increased in both groups during HUT (60°: 39 ± 8 vs. 34 ± 13 bursts/min, *p* < 0.05 from BL). Noradrenaline was higher in white women during 60° HUT (60° HUT: 364 ± 102 vs. 267 ± 89 pg/ml, *p* < 0.05). Direct renin was higher and vasopressin and Δ aldosterone tended to be higher in blacks (BL, direct renin: 12.1 ± 5.0 vs. 14.4 ± 3.7 pg/ml, *p* < 0.05; BL, vasopressin: 0.4 ± 0.0 vs. 1.6 ± 3.6 pg/ml, *p* = 0.065; Δ aldosterone: −0.9 ± 5.1 vs. 3.8 ± 7.5 ng/ml; *p* = 0.069). These results suggest that young normotensive white women may rely on sympathetic neural activity more so than black women who have a tendency to rely on the renal-adrenal system to regulate BP during an orthostatic stress.

## Introduction

Blood pressure (BP) and BP regulation strategies appear to be different between white and black individuals of similar age. For example, the incidence of hypertension is higher among blacks when compared to whites (Burt et al., 1995; Cooper et al., 1996). Previous studies (Calhoun et al., 1993; Calhoun and Mutinga, 1997) have demonstrated that black individuals have greater sympathetic reactivity to stressors than do white. Calhoun et al. (1993) reported that, when comparing responses of normotensive whites and blacks during a cold pressor test, blacks had greater increases in BP and muscle sympathetic nerve activity (MSNA). Additionally, in response to an orthostatic challenge, black individuals have shown an increase in mean BP while other racial groups showed either no change or a decline (Goldstein and Shapiro, 1995; Okada et al., 2012a). More recently, Hinds and Stachenfeld (2010) reported that young black women were more orthostatically tolerant than white women. They showed that baseline haemodynamic and hormonal profiles were comparable; however, when subjected to progressive lower body negative pressure to presyncope, not only were black women more tolerant to the stress, but also had greater increases in noradrenaline. The black women also had a greater change in plasma renin activity from baseline to presyncope, suggesting a larger contribution of the renin-angiotensin-aldosterone system in the regulation of BP.

Additionally, others (Ray and Monahan, 2002) have shown that sympathetic vascular transduction is augmented in young black individuals when compared to white. Ray and Monahan (2002) reported that blacks had a smaller increase in MSNA during lower body negative pressure but with similar increases in forearm vascular resistance as did their white counterparts. Taken together, these studies suggest that greater reactivity to stressors may be one potential explanation for the higher incidence of hypertension in this population. Moreover, the results from these studies indicate that BP regulation strategies between racial groups might be fundamentally different.

Studies like these are critical to further our understanding of racial differences in the regulation of BP. Many of the earlier studies (Calhoun et al., 1993, 1994; Calhoun and Mutinga, 1997; Ray and Monahan, 2002) included both sexes in their investigations, perhaps, making the interpretation of the results difficult because of the dissimilarity in BP regulation strategies between women and men (Jarvis et al., 2010, 2011, 2012; Okada et al., 2012b). There is little information on racial differences in BP control in young white vs. black women. This is a critical issue to address because it has been shown that young black women have a higher prevalence of hypertensive disorders during pregnancy compared to young white women (Tanaka et al., 2007). Based on previous studies in both sexes (Calhoun et al., 1993, 1994; Calhoun and Mutinga, 1997; Ray and Monahan, 2002) and the work of Hinds and Stachenfeld (2010) in women, we sought to examine haemodynamic, neural, and humoral responses to graded head up tilt (HUT) in young, normotensive black and white women. We hypothesized that black women would demonstrate larger increases in MSNA, as well as have greater humoral responses to HUT when compared to white women.

## Methods

### Subjects

Twenty-five (15 white, 10 black) healthy women volunteered for this study. Racial categories were self-reported by the subjects during the screening process, and those with ≥2 races were excluded. Descriptive characteristics of the subjects are outlined in Table 1. Exclusionary criteria included: significant medical history, smoking, recreational drug use, hormonal contraceptive use within the previous 6 months, current or previous hormonal fertility treatments/supplements, and irregular menstrual cycles. All subjects gave written informed consent to participate in the study which was approved by the Institutional Review Boards at the University of Texas Southwestern Medical Center and Texas Health Presbyterian Hospital Dallas. The study followed guidelines set forth in the *Declaration of Helsinki*.

**Table 1:** **Subject characteristics**.

**Variables**	**White (***n*** = **15**)**	**Black (***n*** = **10**)**
Age (yrs)	30 ± 4	32 ± 6
Height (cm)	165.3 ± 5.7	165.9 ± 6.7
Weight (kg)	65.9 ± 9.8	71.5 ± 7.3
BMI (kg/m^2^)	24.2 ± 3.9	26.1 ± 3.2

Values are means ± SD.

### Protocol

All the experiments were performed during the mid-luteal phase of the menstrual cycle (19–22 days after the onset of menstruation, high estrogen and progesterone). For the 2 days prior to the studies the subjects were placed on an isocaloric constant diet consisting of 200 mEq sodium, 100 mEq potassium, and 1000 mg calcium, while water intake was *ad libitum*. The experiment was performed ≥2 h after a light meal and ≥48 h after the last caffeinated or alcoholic beverage was consumed and in a quiet, environmentally controlled laboratory with an ambient temperature of ~25°C. A pregnancy test was performed and showed a negative result prior to testing.

The subject was placed in the supine position and an intravenous catheter was inserted into the antecubital vein of the arm for blood samples. After ≥30 min of quiet rest, baseline haemodynamic measurements were performed. At least 10 min after a satisfactory nerve recording site had been found, baseline data were recorded for 6 min. After that, the subject was passively tilted to 30° HUT for 5 min and then to 60° HUT for 5 min, followed by a 3 min supine recovery (REC) stage. MSNA, heart rate (HR), and BP were recorded continuously, and haemodynamic measurements were repeated at the end of 30 and 60° HUT. Changes from supine baseline and 60° HUT (Δ) were also examined. Forearm blood flow (FBF) was estimated before upright tilt, when the subject was in the supine position.

### Measurements

#### Muscle sympathetic nerve activity

MSNA signals were obtained using the microneurographic technique (Vallbo et al., 1979). Briefly, a recording electrode was placed in the peroneal nerve at the popliteal fossa and a reference electrode was placed subcutaneously 2–3 cm from the recording electrode. The nerve signals were amplified (gain 70,000–160,000), band-pass filtered (700–2000 Hz), full-wave rectified, and integrated with a resistance-capacitance circuit (time constant 0.1 s). Criteria for adequate MSNA recording included: (1) pulse synchrony; (2) facilitation during the hypotensive phase of the Valsalva manoeuvre and suppression during the hypertensive overshoot after release; (3) increases in response to breath holding; and (4) insensitivity to emotional stimuli (Vallbo et al., 1979).

#### Haemodynamics and forearm blood flow

HR was determined from lead II of the electrocardiogram (ECG). Beat-by-beat arterial pressure (systolic BP, SBP; diastolic BP, DBP) was estimated non-invasively by using finger photoplethysmography (Nexfin). Arm cuff (brachial) BP was obtained via electrosphygmomanometry (model 4240, SunTech Medical Instruments Inc., Raleigh, NC, USA). Cardiac output ($\dot{\text{Q}}$c) was obtained via the acetylene (C_2_H_2_) rebreathing technique as described by Triebwasser et al. (1977) which has been previously validated against direct Fick and thermodilution (Jarvis et al., 2007). Total peripheral resistance (TPR) was calculated (TPR = mean BP × 80/$\dot{\text{Q}}$c). FBF was estimated using venous occlusion plethysmography (Hokanson) (Greenfield et al., 1963). Forearm vascular resistance (FVR) was calculated from mean BP divided by FBF.

#### Blood analyses

During baseline we withdrew samples for hormone analyses. In addition, blood samples were also drawn during baseline and at 5 min of 60° upright tilt for assessment of noradrenaline, adrenaline, direct renin, vasopressin, and aldosterone.

### Data analysis

Data were sampled at 625 Hz with a commercial data acquisition system (Biopac System) and analyzed using LabView Software (National Instruments). Beat-by-beat HR was calculated from the R-R interval of the ECG. Beat-by-beat SBP and DBP were estimated from the arterial waveforms. MSNA bursts were identified by a computer program (Cui et al., 2001), and then were confirmed by trained personnel. Burst areas of the integrated neurogram and BP were measured simultaneously on a beat-to-beat basis. Burst frequency was defined as the number of bursts per min and burst incidence was used to normalize burst frequency per 100 heart beats. Total activity was defined as the burst area of the rectified and integrated neurogram. We assigned the largest burst amplitude during baseline a value of 100, which we assigned as our calibration burst (Sugiyama et al., 1996; Halliwill, 2000). Therefore, all other bursts within a testing session were normalized against this value.

#### Assessment of sympathetic vascular transduction

To assess the transduction of sympathetic outflow into vascular resistance we used FVR and MSNA burst frequency and total activity (sympathetic vascular transduction = FVR/MSNA). During graded HUT we used TPR/MSNA burst frequency to estimate vascular transduction.

### Statistical analysis

Data are expressed as means ± SD. A *t*-test was used to compare anthropometric values, blood parameters, FVR, and sympathetic vascular transduction. A two-way repeated measures analysis of variance [group (white, black) × stage (BL, 30° HUT, 60° HUT, REC); ANOVA] was used to examine cardiovascular, MSNA and humoral responses between groups and across stages. We also examined the difference from BL to 60° HUT (Δ) between the racial groups with a two-way repeated measures ANOVA. Tukey's *post-hoc* analysis was used when significance was found. All statistical analyses were performed using SigmaPlot (Systat). A *p*-value of <0.05 was considered statistically significant.

## Results

### Haemodynamics

**Figure 1:**
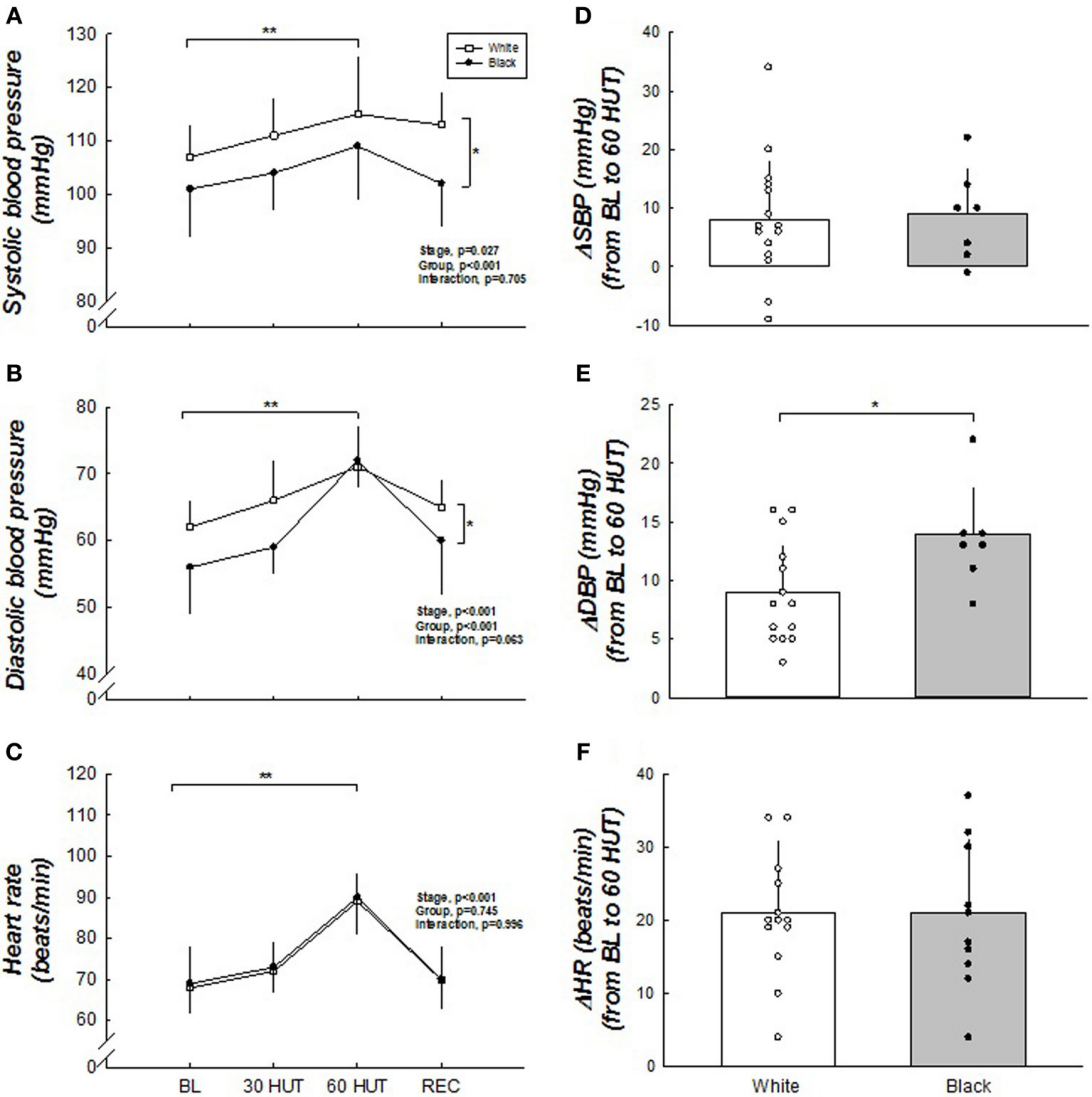
**(A–F)** Blood pressure and heart rate responses to HUT. SBP and DBP were higher in white when compared to black women. Δ DBP was greater in black women. SBP and DBP were higher during 60° HUT when compared to BL. HR was not different between the racial groups but was higher during 60° HUT compared to BL. ^*^Difference between groups, *p* < 0.05. ^**^Difference from BL, *p* < 0.05. For SBP and DBP, *n* = 15 for white and *n* = 7 for black due to two black women becoming presyncopal at 60° HUT and one faulty BP measurement. Therefore, blood pressures were not used for group or individual data from these women in these figures.

Figure 1 shows the brachial BP and HR responses. Both SBP and DBP were higher in the white women compared to the black (both *p* < 0.001). Δ SBP was not different between the racial groups (*p* = 0.50). However, Δ DBP (60° HUT-BL) was greater in black women compared to white (*p* < 0.05). HR was not different between the two groups (*p* = 0.996), nor was Δ HR different between the groups. SBP, DBP, and HR all increased as expected in response to 60° HUT (all *p* < 0.05). Haemodynamic responses are outlined in Table 2. There was no difference between racial groups in haemodynamic variables; however, HUT elicited decreases in cardiac output and stroke volume (both, *p* < 0.05). When cardiac output and stroke volume were normalized to body surface area (cardiac index and stroke index) these relationships did not change. As expected, TPR increased with graded HUT (*p* < 0.05).

**Table 2:** **Haemodynamic variables**.

**Variables**	**White**			**Black**		
	**BL ***n*** = **13****	**30HUT ***n*** = **13****	**60HUT ***n*** = **12****	**BL ***n*** = **10****	**30HUT ***n*** = **9****	**60HUT ***n*** = **6****
Cardiac output (L/min)	4.98 ± 0.82	4.43 ± 0.97	3.76 ± 0.94^†^	5.20 ± 1.01	4.42 ± 0.86	4.19 ± 1.51^†^
Cardiac index (L/min/m^2^)	2.86 ± 0.55	2.55 ± 0.56	2.16 ± 0.52^†^	2.87 ± 0.51	2.42 ± 0.46	2.26 ± 0.87^†^
Stroke volume (ml/beat)	63 ± 11	54 ± 11^†^	40 ± 9^†^	68 ± 12	55 ± 10^†^	43 ± 15^†^
Stroke volume index (ml/beat/m^2^)	36 ± 7	31 ± 6^†^	23 ± 5^†^	38 ± 6	30 ± 5^†^	23 ± 9^†^
Total peripheral resistance (dyne/s/cm^5^)	1267 ± 205	1515 ± 323^†^	1889 ± 390^†^	1129 ± 269	1398 ± 271^†^	1797 ± 574^†^

Values are means ± SD.

† Difference from BL, p < 0.05. The number of subjects is noted for each stage to account for where a cardiac output measurement was not obtained due to a faulty measurement or when a subject experienced symptoms of presyncope and the tilt was terminated.

FBF was not different between the two groups (3.13 ± 1.16 vs. 2.72 ± 0.87 ml/100 ml/min, *p* = 0.349). FVR was also comparable between the groups (28.3 ± 11.1 vs. 29.0 ± 8.6 mmHg/ml/100 ml/min, *p* = 0.867).

### Sympathetic neural activity

**Figure 2:**
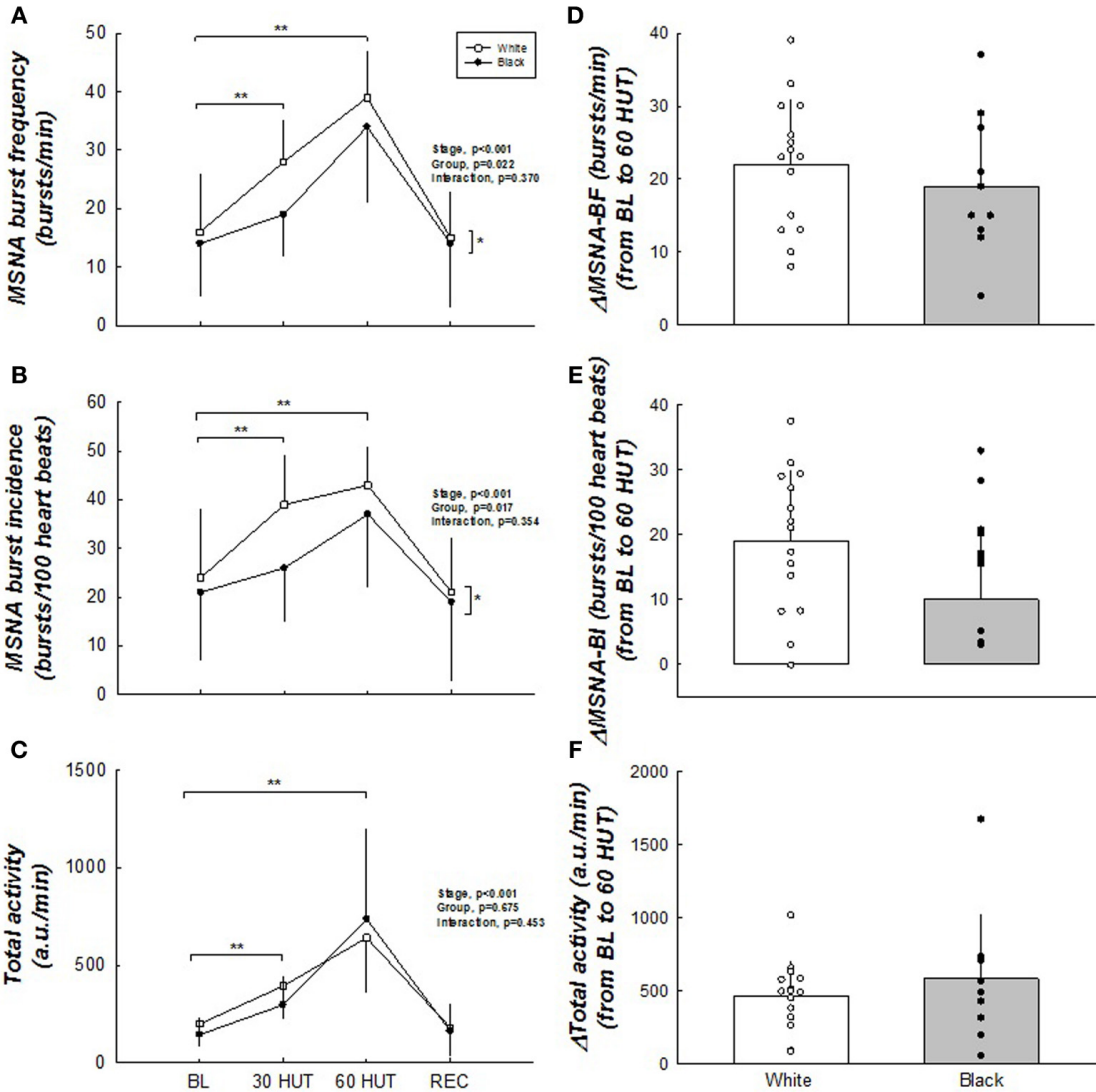
**(A–F)** Muscle sympathetic nerve activity responses to HUT. MSNA burst frequency and burst incidence were higher in white women. Δ MSNA burst frequency tended to be greater in white women. No difference exists between groups with total activity. All MSNA parameters increased upon HUT. ^*^Difference between groups, *p* < 0.05. ^**^Difference from BL, *p* < 0.05. *N* = 15 for white women and *n* = 10 for black women.

Figure 2 illustrates the MSNA responses between the two groups during tilt. MSNA burst frequency and incidence were higher in whites (main effect, both *p* < 0.05). The total activity, however, was not different between the groups (*p* = 0.453). The change from BL to 60° HUT (Δ) was similar between the groups indicating the responses were similar, except that whites tended to have a larger ΔMSNA burst frequency (*p* = 0.066). As expected, all MSNA variables increased in response to graded tilt (all *p* < 0.05).

### Sympathetic vascular transduction

**Figure 3:**
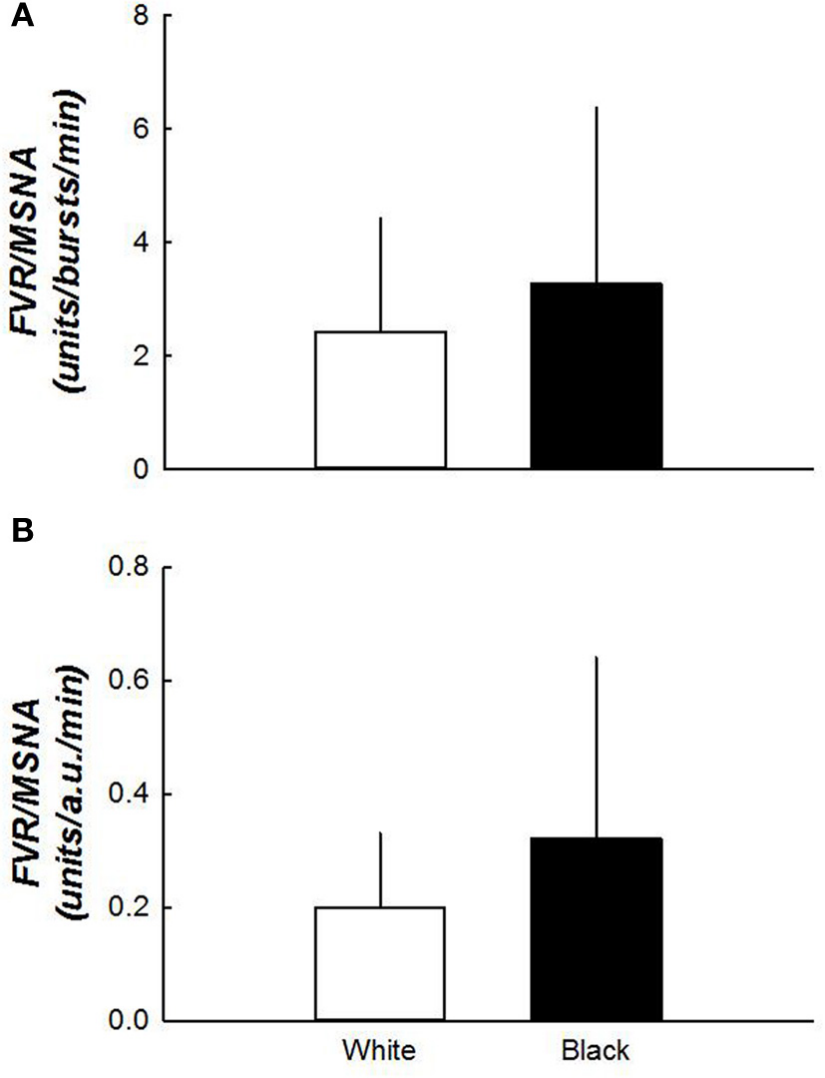
**(A,B)** Sympathetic vascular transduction between white and black women. Vascular transduction in the supine position was not different between the two groups. *N* = 15 for white women and *n* = 10 for black women.

Sympathetic vascular transduction at rest (Figure 3), whether determined from MSNA burst frequency or total activity, was not different between white and black women. Sympathetic vascular transduction (TPR/MSNA burst frequency) during graded HUT tended to be higher in blacks during 30° HUT (59 ± 21 vs. 102 ± 77 units/bursts/min, *p* = 0.067). Estimates for 60° HUT were not possible due to the low number of data points for comparison (*n* = 6 for the black cohort).

### Sex hormones

There was no difference between oestradiol (153 ± 96 vs. 97 ± 63 pg/ml, *p* = NS) and progesterone (8 ± 6 vs. 5 ± 6 ng/ml, *p* = NS) between the two groups.

### Catecholamine and humoral responses to HUT

The catecholamine and humoral responses to 60° HUT are outlined in Table 3. Noradrenaline was similar between the groups during supine baseline; however, white women had a higher noradrenaline concentration at 60° HUT when compared to black women (*p* < 0.05). Black women had higher direct renin when compared to white women (main effect, *p* < 0.05). Vasopressin and Δ aldosterone tended to be higher in blacks (vasopressin, *p* = 0.065; Δ aldosterone, *p* = 0.069). There was no significant difference in adrenaline between the two groups. Moving from supine baseline to 60° HUT induced increases in noradrenaline, adrenaline, and vasopressin (all *p* < 0.05). The Δ for each variable was similar between the two races.

**Table 3:** **Catecholamine and hormonal responses to HUT**.

	**White**			**Black**		
	**BL ***n*** = **15****	**60HUT ***n*** = **15****	**Δ**	**BL ***n*** = **9****	**60HUT ***n*** = **8****	**Δ**
Noradrenaline (pg/ml)	210 ± 87	364 ± 102^†^^*^	154 ± 90	169 ± 50	267 ± 89^†^^*^	105 ± 79
Adrenaline (pg/ml)	16 ± 12	58 ± 33^†^	42 ± 33	12 ± 6	60 ± 59^†^	48 ± 58
Direct renin^**^ (pg/ml)	12.0 ± 5.0	10.7 ± 5.0	−1.3 ± 2.4	14.4 ± 3.7	14.9 ± 6.6	0.8 ± 7.4
Vasopressin (pg/ml)	0.4 ± 0.0	2.1 ± 4.8^†^	1.7 ± 4.8	1.6 ± 3.6	6.8 ± 9.9^†^	5 ± 10
Aldosterone (ng/dl)	6.3 ± 5.7	5.4 ± 6.4	−0.9 ± 5.1	5.3 ± 4.2	9.0 ± 11.0	3.8 ± 7.5

Values are means ± SD.

* Difference between groups, p < 0.05.

** Difference in main effect of race with direct renin.

† Difference from BL, p < 0.05. The number of subjects is noted for each stage to account for where a blood draw was not obtained. For the black cohort, one subject refused the blood draw and the other experienced symptoms of presyncope and the tilt was terminated before the blood draw was complete.

## Discussion

To the best of our knowledge, the current study is one of the first to examine haemodynamic, neural, and humoral responses to graded HUT in a group of young, normotensive white and black women. Previous studies have included mixed sex populations and have indicated that blacks have higher reactivity to a variety of laboratory stressors (Calhoun et al., 1993, 1994; Ray and Monahan, 2002). Given the differences between women and men in the regulation of BP (Jarvis et al., 2010, 2011, 2012; Okada et al., 2012b), it may be difficult to interpret these studies. The principal findings from the current study are: (1) white women demonstrated higher MSNA and upright catecholamines along with higher concomitant BP than black women; (2) black women demonstrated augmented Δ DBP in response to HUT and a tendency toward enhanced renal-adrenal responses compared to white women; and (3) the transduction of sympathetic outflow into vascular resistance during HUT tended to be enhanced in blacks. These results suggest that white and black women might have different strategies in the regulation of BP. White women may rely more on neurally mediated increases (e.g., MSNA burst frequency) to maintain BP more so than black women.

### Sympathetic neural control during HUT between racial groups

Contrary to our hypothesis, white women displayed higher BP, had higher MSNA, and tended to have a greater MSNA response (Δ MSNA) to HUT than did the black women. Consistent with the higher BP and MSNA, the white women demonstrated an elevated noradrenaline response during HUT compared to their black counterparts. The higher noradrenaline concentration in the white cohort is consistent with a previous report that showed noradrenaline clearance was higher in black males when compared to white (Ziegler et al., 1991). However, our findings are in contrast to a previous report by Hinds and Stachenfeld (2010) who reported blacks having a greater increase in noradrenaline. The difference between our study and theirs might be explained by the time periods chosen for analysis. That is, our subjects remained in the upright posture for 5 min, whereas Hinds and Stachenfeld (2010) performed lower body negative pressure until a loss of haemodynamic stability (presyncope). Additionally, we excluded women who were on hormonal contraceptives within the previous 6 months and all subjects were tested during the mid-luteal phase of the menstrual cycle. We found that black women demonstrated a larger Δ DBP in response to HUT, indicating they may be more “reactive” to an orthostatic stress and/or rely on non-adrenergic inputs to increase BP. Consistent with the findings in the current study, Okada et al. (Okada et al., 2012a) recently reported that the MSNA responses were larger in elderly whites (both males and females) and that blacks demonstrated a greater increase in DBP in response to 60° HUT. Ray and Monahan (2002) also reported that during progressive lower body negative pressure—another method to unload arterial baroreceptors—whites demonstrated greater increases in MSNA. They, however, reported similar mean BP for the mixed-sex groups during baseline and in response to lower body negative pressure (Ray and Monahan, 2002).

While there have been consistent reports (Ray and Monahan, 2002; Franke et al., 2004; Okada et al., 2012a) that whites demonstrate a greater neural response to baroreceptor unloading, few have tried to explain the mechanism. Okada et al. (2012a) outlined an interpretation of Kienbaum et al.'s (2001) work as it applied to elderly whites vs. blacks. The current study might extend this interpretation. Kienbaum et al. (2001) assert that there are two sites that modulate sympathetic responses elicited by the arterial baroreceptors. Site 1 is a gated “yes/no” response—that is, it determines whether the burst occurs. Site 2 determines the strength of the response—that is, it determines the amplitude or total MSNA activity based on feedback from the other areas located within the central nervous system, as well as the periphery (Kienbaum et al., 2001). Okada et al. (2012a) suggested that in elderly individuals, blacks might have a blunted site 2. This is corroborated by Ray and Monahan (2002) that report total MSNA (%) increased to a greater degree in whites during −40 mmHg lower body negative pressure when compared to blacks. Findings from the current study provide evidence to suggest that young black and white women might have a similar site 2 but that young blacks may have a blunted site 1. Additionally, these findings might indicate that sex differences within blacks might exist in the modulation of MSNA from these sites. Therefore, we might conclude that the threshold for whether a burst will occur may be higher for young black women or the sensitivity is blunted. We might also conclude that differences in the modulation of MSNA from site 1 (whether a burst will occur) differ between black women and men. Consequently, this may require blacks to rely on other strategies to effectively maintain BP.

We found that at rest sympathetic vascular transduction was similar between whites and blacks. However, during 30° HUT there was a trend toward a greater degree of transduction in the black cohort. This finding is consistent with other reports examining racial differences. For example, Ray and Monahan (2002) reported greater sympathetic vascular transduction in blacks during progressive lower body negative pressure. It has also been demonstrated that black individuals had lower forearm vascular conductance during progressive lower body negative pressure, despite the racial groups having similar baseline values (Franke et al., 2004). Together, these suggest that blacks may be more “responsive” than the white cohort during baroreceptor unloading.

Others (Stein et al., 2000) have shown that blacks were more reactive to infusions of phenylephrine, an α_1_-adrenergic agonist, when assessed by forearm vascular resistance. They also demonstrated similar catecholamine concentrations between the racial groups but that the blacks were more responsive to that concentration. This is consistent with our findings as despite a lower neurotransmitter concentration, the black women tended to have a greater transduction compared to the white women. This suggests that greater α_1_-adrenergic sensitivity and/or enhanced receptor density may exist. Additionally, it is likely that a non-adrenergic humoral mechanism also played a role in the trend toward vascular responsiveness differences in our subjects. For example, renin—a potent vasoconstrictor—was higher in the black women.

### Renal-adrenal responses during HUT between racial groups

We found that direct renin was higher in the black cohort and that vasopressin and Δaldosterone also tended to be greater. Consistent with others (Hinds and Stachenfeld, 2010), plasma renin activity has been shown to be increased to a larger extent in black women exposed to lower body negative pressure when compared to a white cohort. While direct renin is a precursor to angiotensin II, which serves as a sympathoexcitatory agent, we did not find that MSNA was concomitantly augmented in blacks. This might suggest that there are dissimilarities in the distribution of sympathetic outflow. That is, white women might demonstrate a larger increase in MSNA and black women might demonstrate a larger increase in renal sympathetic nerve activity in order to preserve or maintain BP during an orthostatic stress. The line of reasoning to support this is that an increase in renal sympathetic activity stimulates β_1_-adrenergic receptors in the juxtaglomelular cells, which stimulates the release of renin. Additionally, angiotensin II stimulates the adrenal cortex to release aldosterone. Therefore, the renal-adrenal system may play a larger role in BP regulation in black individuals. It is possible that this system is enhanced to offset the deficit in the neural control of MSNA (e.g., site 1), so that BP can be effectively maintained. Alternatively, neural control of MSNA may be attenuated due to the overactivity of the renal-adrenal contributions to BP control.

### Limitations

First, sympathetic neural activity was assessed via microneurography. It is plausible that there are dissimilarities in the distribution of neural responses to other vascular beds such as the splanchnic and renal beds. Thus, we used products of the renin-angiotensin-aldosterone system as a proxy for this distribution which may not be accurate. However, given the potential differences in BP regulation strategies between these two groups we do not feel this limitation alters our conclusions. A second limitation might be with how total activity is calculated and how these values may be over-estimated in the black women. Baseline MSNA is used to detect the calibration signal. We found the height of the calibration signal was roughly half in black women compared to the white. This may have resulted in an over-estimation of total activity during HUT in the black women. Third, we obtained nerve recordings in the peroneal nerve but used resistance calculated in the forearm. While this design is not perfect, we feel this method is defensible as others (Rea and Wallin, 1989) reported that during progressive lower body negative pressure MSNA, measured in both the arm and leg, had similar baselines and increased similarly in response to the stress. While there may be differences in vascular resistance, likely due to limb dependency induced venoarteriolar reflex, we believe this would underestimate vascular transduction rather than overestimate it. Moreover, this technique was uniformly applied to all subjects such that any error in this technique would be uniformly applied. Lastly, *post-hoc* analysis indicated that we were underpowered to detect a difference in some of the variables (MSNA, for example). Thus, we are cautious in outlining any definitive conclusions. However, we feel that these trends may be of potential significance and help our understanding of some of the racial differences observed in cardiovascular regulation.

## Author contributions

Sara S. Jarvis contributed to (1) conception and design of the experiments; (2) collection, analysis and interpretation of data; and (3) drafting the article and revising it critically for important intellectual content. Shigeki Shibata contributed to (1) conception and design of the experiments; (2) collection, analysis and interpretation of data; and (3) revising the article for important intellectual content. Yoshiyuki Okada contributed to (1) conception and design of the experiments; (2) collection, analysis and interpretation of data; and (3) drafting the article and revising it critically for important intellectual content. Benjamin D. Levine contributed to (1) conception and design of the experiments; (2) collection, analysis and interpretation of data; and (3) revising the article for important intellectual content. Qi Fu contributed to (1) conception and design of the experiments; (2) collection, analysis and interpretation of data; and (3) drafting the article and revising it critically for important intellectual content. All authors approved the final version of the manuscript.

## Funding

This study was supported by the National Institutes of Health grant R21 HL088184 (Qi Fu) and the American Heart Association postdoctoral fellowship grant 10POST362001 (Sara S. Jarvis).

### Conflict of interest statement

The authors declare that the research was conducted in the absence of any commercial or financial relationships that could be construed as a potential conflict of interest.
